# A Cyclic Altered Peptide Analogue Based on Myelin Basic Protein 87–99 Provides Lasting Prophylactic and Therapeutic Protection Against Acute Experimental Autoimmune Encephalomyelitis

**DOI:** 10.3390/molecules23020304

**Published:** 2018-01-31

**Authors:** Mary Emmanouil, Vivian Tseveleki, Iro Triantafyllakou, Agathi Nteli, Theodore Tselios, Lesley Probert

**Affiliations:** 1 Laboratory of Molecular Genetics, Hellenic Pasteur Institute, 127 Vasilissis Sophias Ave., 11521 Athens, Greece; emmanouilm@pasteur.gr (M.E.); tseveleki@gmail.com (V.T.); 2 Department of Chemistry, University of Patras, 26504 Patras, Greece; irotriant@gmail.com (I.T.); anteli@upatras.gr (A.N.)

**Keywords:** myelin basic protein (MBP), experimental autoimmune encephalomyelitis (EAE), multiple sclerosis (MS), altered peptide ligands (APLs), cyclic peptides

## Abstract

In this report, amide-linked cyclic peptide analogues of the 87–99 myelin basic protein (MBP) epitope, a candidate autoantigen in multiple sclerosis (MS), are tested for therapeutic efficacy in experimental autoimmune encephalomyelitis (EAE). Cyclic altered peptide analogues of MBP_87–99_ with substitutions at positions 91 and/or 96 were tested for protective effects when administered using prophylactic or early therapeutic protocols in MBP_72–85_-induced EAE in Lewis rats. The Lys^91^ and Pro^96^ of MBP_87–99_ are crucial T-cell receptor (TCR) anchors and participate in the formation of trimolecular complex between the TCR-antigen (peptide)-MHC (major histocompability complex) for the stimulation of encephalitogenic T cells that are necessary for EAE induction and are implicated in MS. The cyclic peptides were synthesized using Solid Phase Peptide Synthesis (SPPS) applied on the 9-fluorenylmethyloxycarboxyl/tert-butyl Fmoc/tBu methodology and combined with the 2-chlorotrityl chloride resin (CLTR-Cl). Cyclo(91–99)[Ala^96^]MBP_87–99_, cyclo(87–99)[Ala^91,96^]MBP_87–99_ and cyclo(87–99)[Arg^91^, Ala^96^]MBP_87–99_, but not wild-type linear MBP_87–99_, strongly inhibited MBP_72–85_-induced EAE in Lewis rats when administered using prophylactic and early therapeutic vaccination protocols. In particular, cyclo(87–99)[Arg^91^, Ala^96^]MBP_87–99_ was highly effective in preventing the onset and development of clinical symptoms and spinal cord pathology and providing lasting protection against EAE induction.

## 1. Introduction

Multiple sclerosis (MS) is a chronic immune-mediated disease of the central nervous system (CNS) causing demyelination and progressive neuronal damage [1]. Experimental autoimmune encephalomyelitis (EAE) is a well-characterized experimental model for MS that is induced by immunization of susceptible animal strains with myelin proteins or immunodominant peptide epitopes from proteins such as myelin basic protein (MBP), myelin oligodendrocyte glycoprotein (MOG), and proteolipid protein (PLP). EAE recapitulates the autoimmune T cell-mediated attack on CNS myelin that is believed to underlie MS pathogenesis [2], and is a valuable in vivo system for the design and preclinical testing of experimental therapeutics intended for MS [3]. MBP is a candidate autoantigen in MS [4,5,6,7,8], with the MBP_86–95_ peptide representing an immunodominant epitope centre for human autoantibody binding and T-cell recognition [9], and this epitope has been used for the development of selective peptide-specific immunotherapeutics for MS [10,11,12,13,14].

Altered peptide ligands (APLs) based on the MBP_86–95_ core epitope, in which primary T-cell receptor (TCR) anchors Lys and Pro at positions 91 and 96, are replaced with Arg and/or Ala, respectively, protecting mice against EAE [15,16] and induce differential activation of human T-cell lines [11]. APL stimulate differential T-cell receptor (TCR) signaling and cellular responses compared to wild-type peptides, and can antagonize [17], anergize [18] or partially activate [19] T cells. They can deviate immune responses away from autoimmune Th1 responses towards allergic Th2 responses, and thereby ameliorate EAE [20,21]. Clinical trials using such APL in MS patients provided important proof-of-principle that immune deviation of MBP-specific T-cell responses impacts disease severity, but studies were halted because of safety concerns associated with immune activation [12,13]. A novel alternative approach to the development of peptide therapeutics for autoimmune disease is the design and synthesis of cyclic peptides. Peptide cyclization restricts the conformational flexibility of linear counterparts and results in increased metabolic stability and receptor selectivity, thereby providing a better pharmacological profile, and possibly the ability to alter TCR signaling outcomes [22]. The design of cyclic peptide analogues by connecting residues that are least important for activity, without causing drastic structural changes in bioactive conformation of the peptide, results in a rigid geometry and better bioavailability of cyclic peptides compared to the linear ones. Over the last years our group has been involved in the rational design and synthesis of bioactive mutated cyclic peptides based on the MBP_83−99_ core epitope [23,24,25,26,27,28], and incorporating previous knowledge concerning derived APLs that exhibit immune-modulatory function [6,11,12].

We chose the MBP_87–99_ sequence as a model for the rational design of cyclic APL design because it is a candidate auto-antigen in MS, and induces T-cell responses and EAE in Lewis rats and SJL mice [24,28]. Peptide cyclization is achieved between the N and C terminal or the side chain amino group of Lys^91^ and the C terminal via an amide bond, while the Lys and Pro at positions 91 and 96, respectively, are replaced with Arg and/or Ala [29,30]. We show previously that cyclic MBP_87–99_ analogues bind efficiently to HLA-DR4 and are more stable to hydrolysis by lysosomal enzymes and cathepsin D, compared to their linear counterparts [25,26], and that the three cyclic mutated analogues of MBP_87–99_ used here, specifically cyclo(91–99)[Ala^96^]MBP_87–99_ [24], cyclo(87–99)[Ala^91^, Ala^96^]MBP_87–99_ [26], and cyclo(87–99)[Arg^91^, Ala^96^]MBP_87–99_ [24], ameliorate EAE in Lewis rats when co-administered with MBP_72–85_ during immunization. However, the mechanism of action of cyclic peptide analogues, whether they protect against EAE passively by competing with MBP_72–85_ for MHC class II binding, actively through induction of a Th1/Th2 shift or regulatory T-cell differentiation, or a different mechanism, remains to be determined. In the present study, we investigated the ability of MBP_87–99_ cyclic mutated analogues to protect against EAE induced in Lewis rats by MBP_72–85_ when administered separately from the immunizing antigen using prophylactic (vaccination) and therapeutic protocols, thereby minimizing the possibility of direct competition between MBP_72–85_ and cyclic MBP_87–99_ analogues for MHC class II binding. We also investigated whether immunization with cyclic MBP_87–99_ analogues led to formation of immunological memory that allowed protection to persist over time.

## 2. Results

### 2.1. Solid-Phase Peptide Synthesis and Cyclization of the Mutated Peptide Analogues

**Scheme 1:**
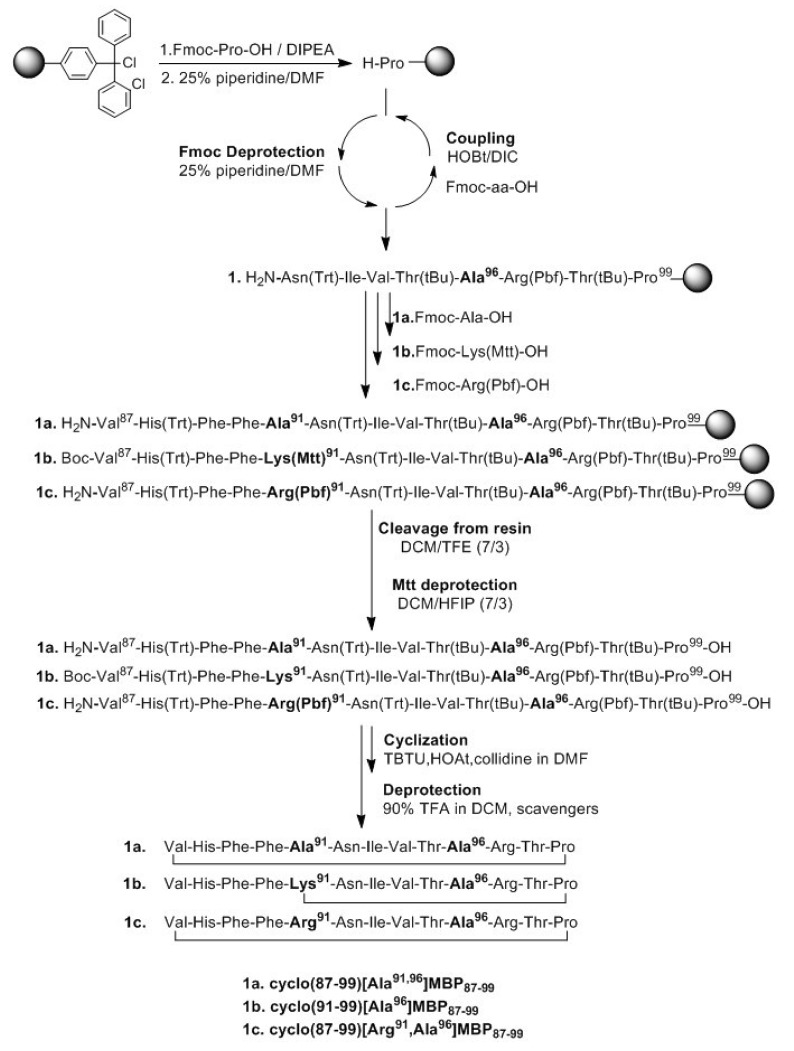
Synthetic procedure of cyclic peptide analogues of myelin basic protein (MBP).

The synthetic procedure of linear peptide analogues and subsequently their cyclization is presented in Scheme 1. The first *N^α^*-9-fluorenylmethyloxycarboxyl (Fmoc) protected amino acid, was esterified to the 2-chlorotrytil chloride (CLTR) resin in the presence of *N*,*N*-diisopropylethylamine (DIPEA), followed by Fmoc deprotection. The peptide bonds were accomplished using 1-hydroxybenzotriazole (HOBt) and *N*,*N′*-diisopropylcarbodiimide (DIC) as coupling reagents. The side chains of amino acids were protected as follows: Trt for Asn, His, Pbf for Arg and tBu for Thr. In the case of cyclo(91–99)[Ala^96^] MBP_87–99_, in which the side chain amine group of Lys at position 91 and C-terminal carboxyl group formed an amide bond, the 4-methyltrityl (Mtt) group was used for protection. This group was removed upon treatment of protected peptide-resin with the splitting mixture dichloromethane (DCM)/1,1,1,3,3,3-hexafluoro-2-propanol (HFIP) (7/3) that used to cleave the peptide from the resin. The use of the 2-chlorotrityl chloride resin, as well as of mild cleaving conditions, allowed the protected peptides to release from the resin and the subsequent cyclization of the desired protected peptides. Cyclization was achieved using *O*-(benzotriazol-1-yl)-*N*,*N*,*N′*,*N′*-tetramethyluronium tetrafluoroborate (TBTU), 1-hydroxy-7-azabenzotriazole (HOAt) and 2,4,6-collidine (2,4,6-trimethylpyridine). The final deprotection of each peptide was achieved using trifluoroacetic acid (TFA) in DCM in the presence of scavengers. The purification was achieved using semi preparative, reverse phase high performance liquid chromatography (RP-HPLC) while the peptide purity was assessed by analytical RP-HPLC and by electron spray ionization mass spectrometry (ESI-MS; Supplementary Materials).

### 2.2. Prophylactic or Early Therapeutic Delivery of Cyclic Mutated MBP_87–99_ Analogues Ameliorates MBP_72–85_-EAE

**Figure 1:**
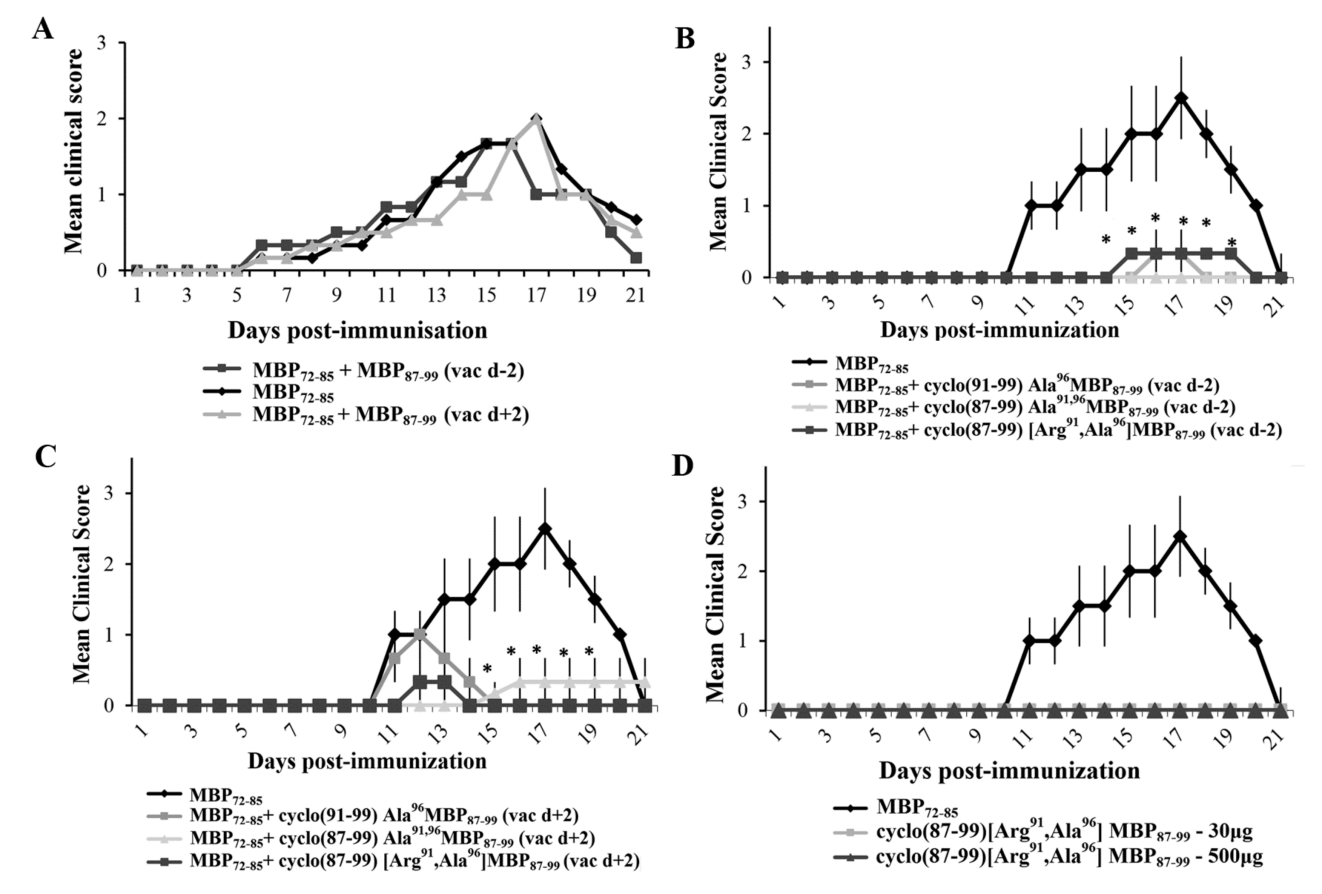
Administration of cyclic myelin basic protein (MBP) 87–99 peptide analogues using prophylactic or therapeutic protocols ameliorates the development of MBP_72–85_-induced experimental autoimmune encephalomyelitis (EAE) in female Lewis rats. (**A**) Mean clinical scores of MBP_72–85_-EAE in groups of rats that were treated subcutaneously (s.c.) with wild type linear MBP_87–99_, as control, either 2 days before (prophylactic) or 2 days after (therapeutic) immunization with MBP_72–85_ (30 μg) for the induction of EAE (*n* = 3 for each group); (**B**,**C**) Mean clinical scores of MBP_72–85_-EAE in groups of rats that were treated s.c. with either cyclo(91–99)[Ala^96^]MBP_87–99_, cyclo(87–99)[Ala^91,96^]MBP_87–99_ or cyclo(87–99)[Arg^91^, Ala^96^]MBP_87–99_ (500 μg), either two days before (**B**) or two days after (**C**) immunization with MBP_72–85_ (30 μg) for the induction of EAE (n = 3 for each group); (**D**) Mean clinical scores of EAE in groups of rats that were immunized with MBP_72–85_ or cyclo(87–99)[Arg^91^, Ala^96^]MBP_87–99_ (at 30 μg and 500 μg), emulsified in complete Freund’s adjuvant (CFA) for the induction of EAE (*n* = 3 for MBP_72–85_; *n* = 3 for cyclic analogue). Data are from one (**A**,**D**, and experiments using cyclo(91–99)[Ala^96^]MBP_87–99_ and cyclo(87–99)[Ala^91,96^]MBP_87–99_), or one representative (experiment 1) of three (experiments using cyclo(87–99)[Arg^91^, Ala^96^]MBP_87–99_), independent experiments (total animals used: experiment 1, *n* = 24; experiment 2, *n* = 18; experiment 3, *n* = 12). Statistical significance after multigroup comparisons, using the Mann–Whitney test, is shown (*, *p* < 0.05).

To test the ability of the cyclic altered MBP_87–99_ analogues to ameliorate EAE when administered in prophylactic or early therapeutic protocols, we administered them to Lewis rats s.c., two days before or two days after, respectively, immunization with MBP_72–85_ for the induction of EAE. First we excluded the possibility that wild-type linear peptide, MBP_87–99_, altered the development of EAE. Administration of linear MBP_87–99_ (500 μg) two days before, or two days after, immunization with MBP_72–85_ for the induction of EAE, did not alter the development of acute monophasic EAE compared to nontreated animals (Figure 1A). In contrast, administration of the cyclic altered analogues cyclo(91–99)[Ala^96^]MBP_87–99_, cyclo(87–99)[Ala^91,96^]MBP_87–99_ or cyclo(87–99)[Arg^91^, Ala^96^]MBP_87–99_ (500 μg), either two days before, or two days after, immunization with MBP_72–85_ all markedly ameliorated the development of EAE (Figure 1B,C). We selected one of the cyclic analogues, cyclo(87–99)[Arg^91^, Ala^96^]MBP_87–99_, for further studies (Table 1). To exclude the possibility that cyclo(87–99)[Arg^91^, Ala^96^]MBP_87–99_ has EAE-inducing potential, we immunized mice with 30 μg or 500 μg analogue using the EAE immunization protocol, and confirmed that neither amount induced EAE (Figure 1D).

**Figure 2:**
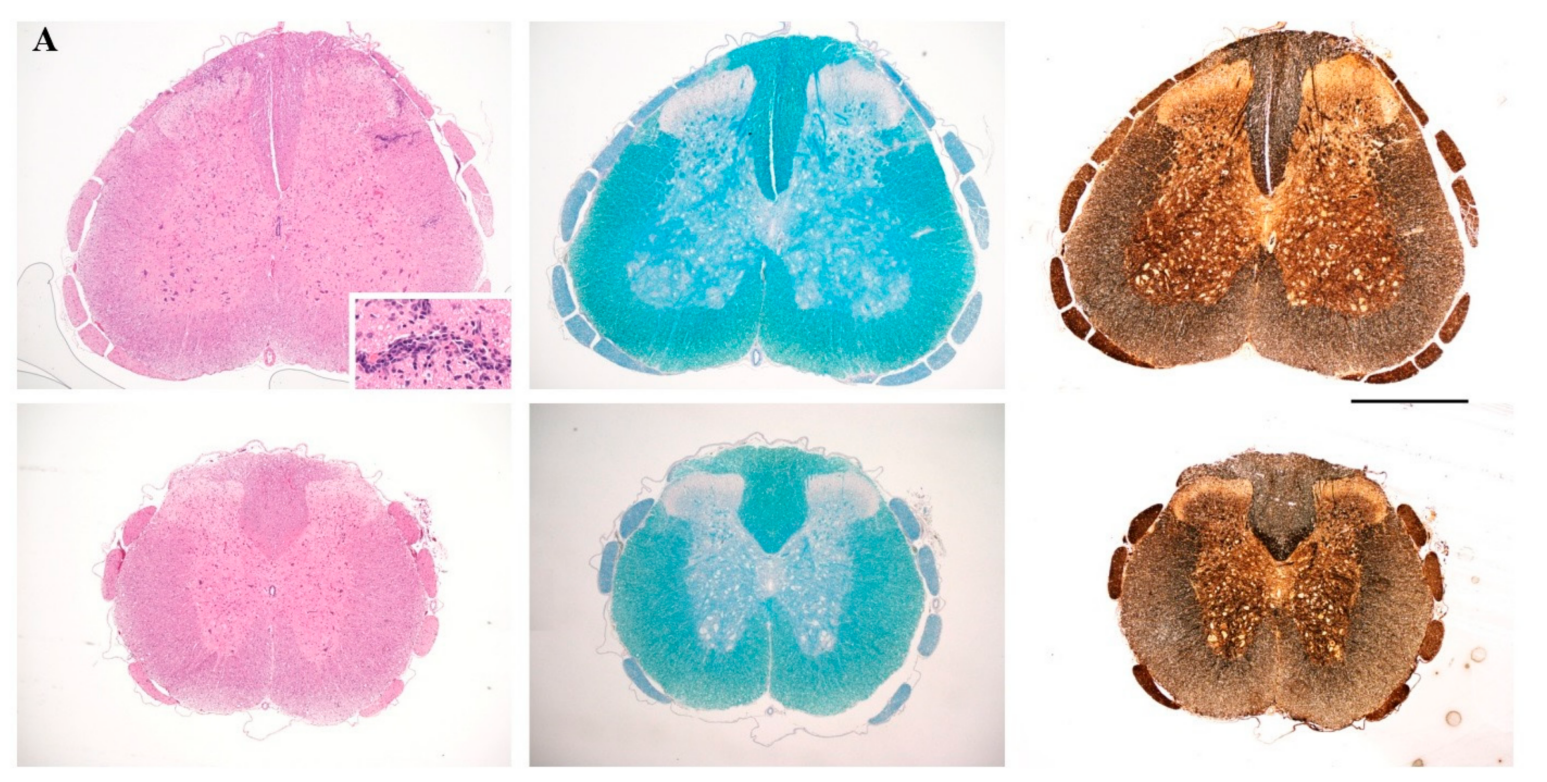

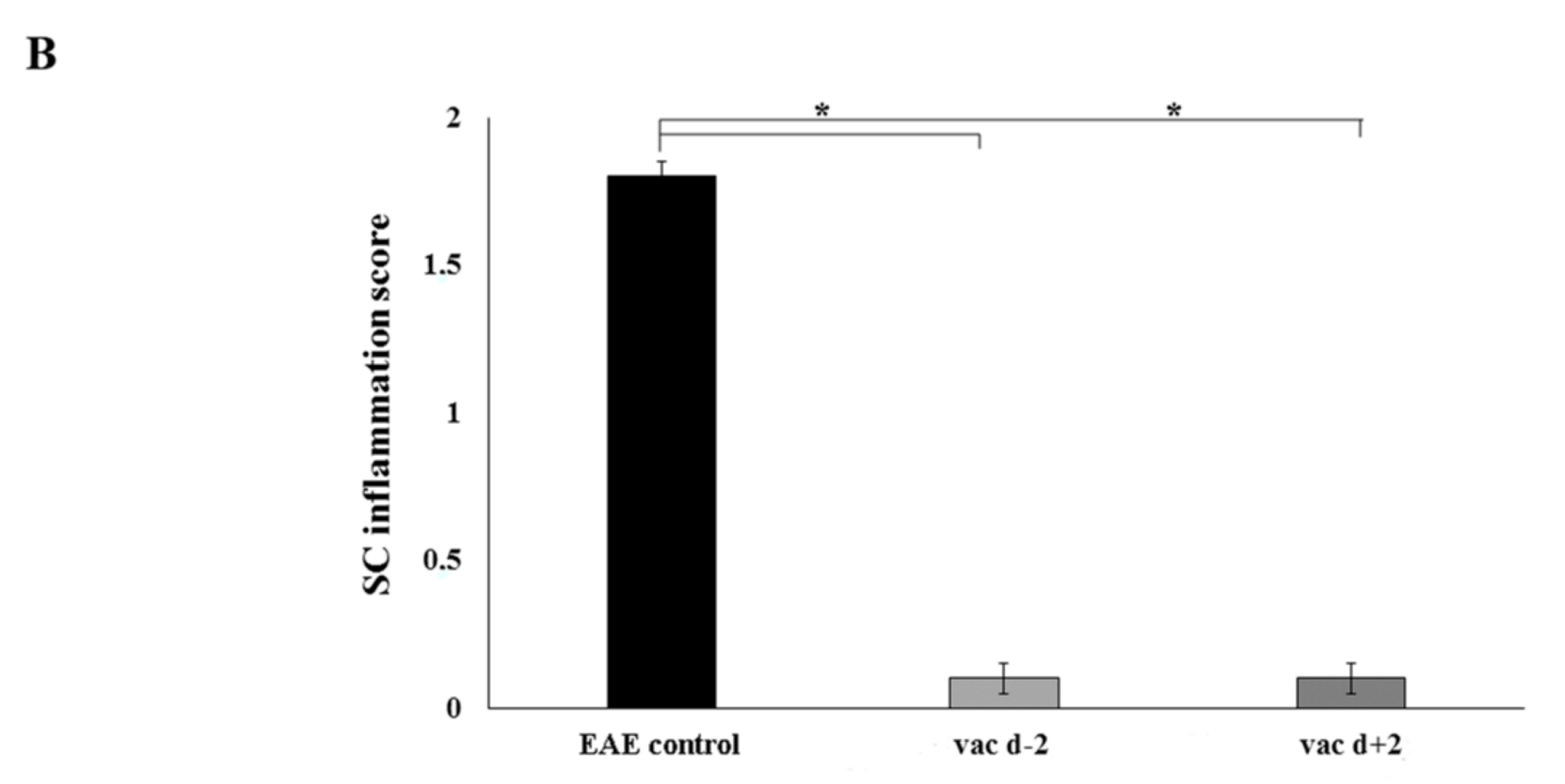
(**A**) Neuropathological analysis of spinal cord sections taken on day 17 following immunization, from representative nonvaccinated control rats (top) and rats vaccinated, two days before (prophylactic) induction of myelin basic protein 72–85 epitope (MBP_72–85_)-induced experimental autoimmune encephalomyelitis (EAE), with cyclo(87–99)[Arg^91^, Ala^96^]MBP_87–99_ (bottom). Sections were stained with H&E for inflammation (left panels), Luxol Fast Blue for myelin (central panels), and Bielschowsky’s silver stain for axons (right panels). Inset shows inflammatory infiltrates in the spinal cord parenchyma. Representative sections from 1 animal per group are shown (*n* = 3 for each group). Scale bar 1 mm; (**B**) Semi-quantitative representation of spinal cord (SC) inflammation in nonvaccinated EAE control rats (EAE control), rats vaccinated with cyclo(87–99)[Arg^91^, Ala^96^]MBP_87–99_, 2 days before (vac d − 2) and 2 days after (vac d + 2) induction of MBP_72–85_-EAE. Pathological changes of spinal cord sections were scored as inflammatory infiltrates/mm^2^ of tissue for the three groups of rats (*n* = 3 for each group). Statistical significance after multi-group comparisons, using the Mann–Whitney test, is shown (*, *p* < 0.05).

To investigate whether amelioration of the clinical symptoms of EAE by the cyclic peptide analogues was associated with reduction of neuropathology, we chose one of the analogues, cyclo(87–99)[Arg^91^, Ala^96^]MBP_87–99_, and administered it to MBP_72–85_-EAE rats using prophylactic and early therapeutic protocols. Rats were sacrificed on day 17, at the peak of clinical symptoms in the untreated control group (Figure 1), and spinal cords analyzed by standard histopathological techniques. Untreated EAE rats showed characteristic features of MBP_72–85_-EAE in Lewis rats; infiltration of the spinal cord parenchyma by immune cells, without white matter demyelination or axonal damage (Figure 2A, upper row). In contrast, treated rats showed no infiltration of the spinal cord parenchyma and few T cells in the meninges (Figure 2A, bottom row).

### 2.3. Cyclic Altered MBP_87–99_ Analogues Induce T Cell Proliferation Responses

**Figure 3:**
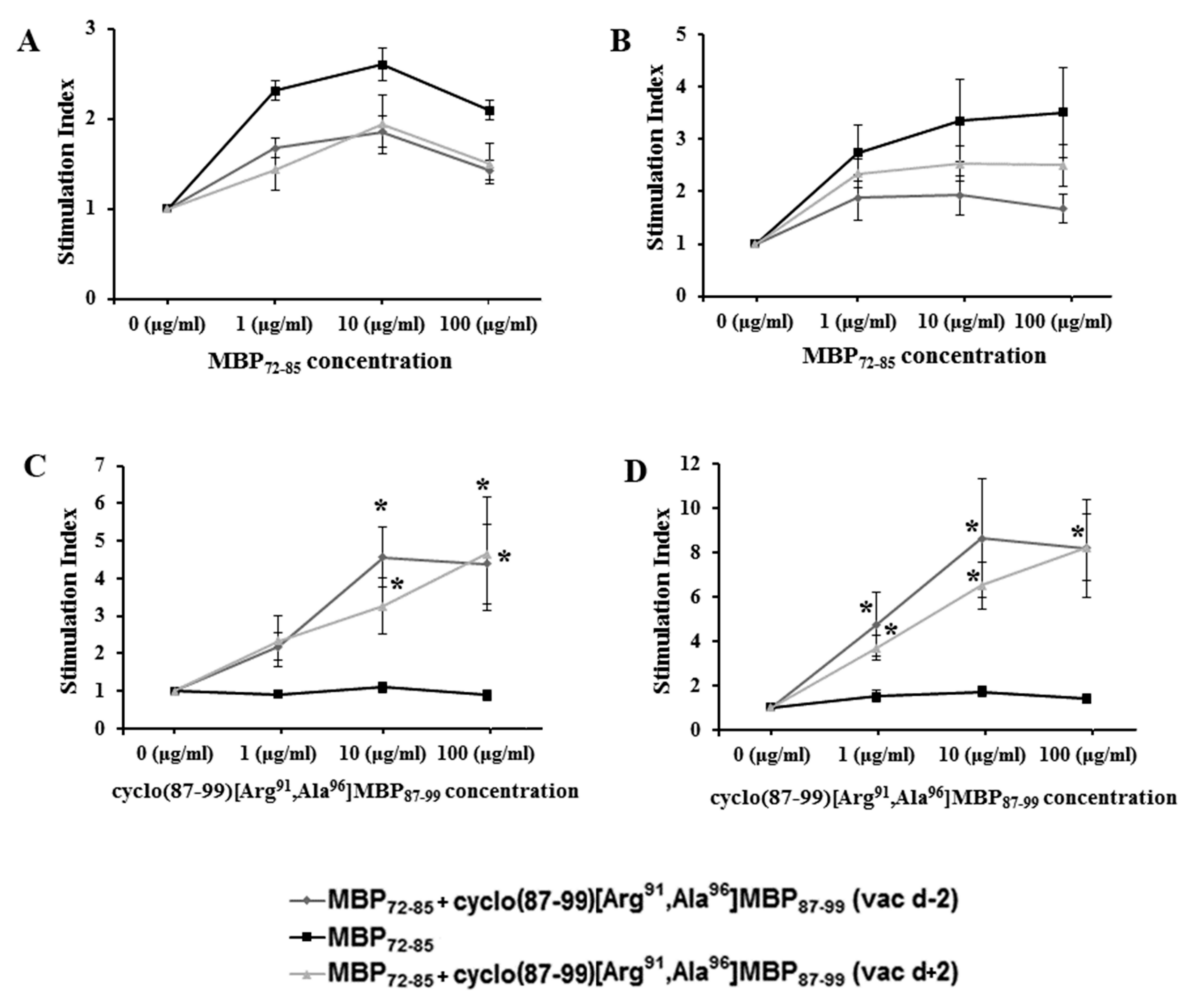
Proliferation of draining lymph node cells (**A**,**C**) and splenocytes (**B**,**D**) from control vehicle-treated rats, and rats treated with cyclic myelin basic protein (MBP) peptide analogue, cyclo(87–99)[Arg^91^, Ala^96^]MBP_87–99_, isolated 10 days post-immunization with MBP_72–85_ for the induction of experimental autoimmune encephalomyelitis (EAE). Cells were stimulated ex vivo with increasing concentrations of linear MBP_72–85_ agonist (**A**,**B**) or cyclo(87–99)[Arg^91^, Ala^96^]MBP_87–99_ peptide (**C**,**D**) (*n* = 4 per group). Data are from one (**A**,**B**), or one representative of two (**C**,**D**) independent experiments. Statistical significance after pair-wise comparisons (using Student’s *t*-test) of each experimental group with the control untreated, MBP_72–85_-induced EAE group is shown (*, *p* < 0.05).

To investigate whether rats treated with cyclo(87–99)[Arg^91^, Ala^96^]MBP_87–99_ mounted antigen-specific T-cell responses, we measured ex vivo T-cell proliferation responses to MBP_72–85_ agonist and the cyclic MBP_87–99_ analogues in DLN cells and splenocytes isolated on day 10 p.i. from EAE rats from the different treatment groups. As expected, control vehicle-treated rats immunized with MBP_72–85_ for the induction of EAE, but not treated with cyclic analogue, showed T-cell proliferation responses to MBP_72–85_ agonist (Figure 3A,B), but not to cyclo(87–99)[Arg^91^, Ala^96^]MBP_87–99_ (Figure 3C,D). In contrast, rats that were treated prophylactically or therapeutically with cyclic analogue, showed strong T-cell responses to the analogue (Figure 3C,D) and mild responses to the MBP_72–85_ agonist (Figure 3A,B). These results suggest that T cells specific for cyclo(87–99)[Arg^91^, Ala^96^]MBP_87–99_ do not inhibit T-cell responses to MBP_72–85_ agonist, when they are induced prior to or two days after immunization with the agonist.

### 2.4. Prophylactic Treatment with cyclo(87–99)[Arg^91^, Ala^96^]MBP_87–99_ Provides Lasting Protection Against MBP_72–85_-Induced EAE

**Figure 4:**
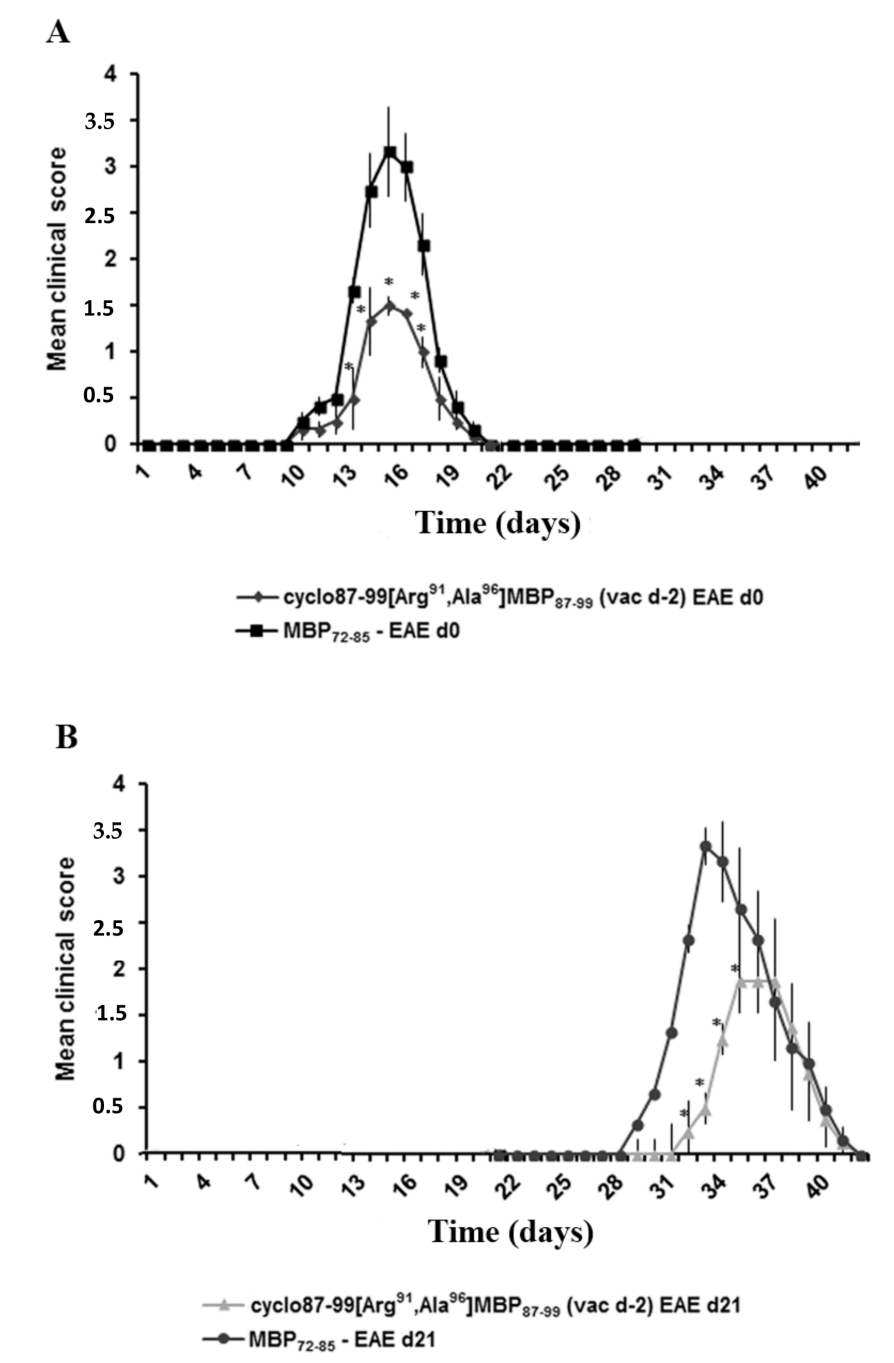
Prophylactic administration of cyclic myelin basic protein (MBP) peptide analogue, cyclo(87–99)[Arg^91^, Ala^96^]MBP_87–99_, provides lasting protection against the development of MBP_72–85_-induced experimental autoimmune encephalomyelitis (EAE) in female Lewis rats. Mean clinical scores of MBP_72–85_-induced EAE in groups of rats that were treated prophylactically subcutaneously (s.c.) with cyclo(87–99)[Arg^91^, Ala^96^]MBP_87–99_ (500 μg), either 2 days, or 23 days before immunization for MBP_72–85_-EAE. A first group of untreated and treated rats (*n* = 6 per group) was immunized with MBP_72–85_ for the induction of EAE on day 2 relative to treatment with cyclic analogue (day 0 on graph) (**A**), and a second group (*n* = 4 per group) on day 23 relative to treatment with cyclic analogue (day 21 on graph) (**B**). Data are from one experiment. Statistical significance after pair-wise comparisons between groups of rats (using Mann–Whitney test) is shown (*, *p* < 0.05).

To determine the potential of cyclo(87–99)[Arg^91^, Ala^96^]MBP_87–99_ treatment to provide long-term protection against MBP_72–85_-induced EAE, we administered the cyclic analogue according to the prophylactic protocol. In one group of treated rats, EAE was induced as usual, two days later, by immunization with MBP_72–85_. As expected, rats treated with cyclo(87–99)[Arg^91^, Ala^96^]MBP_87–99_ showed markedly reduced clinical symptoms compared to untreated control rats (Figure 4A). In a second group of treated rats, EAE was induced 23 days later, by immunization with MBP_72–85_. Again, rats treated with cyclo(87–99)[Arg^91^, Ala^96^]MBP_87–99_ showed markedly reduced clinical symptoms compared to untreated control rats immunized with MBP_72–85_ for the induction of EAE on day 23 of analogue treatment (Figure 4B). These results show that prophylactic treatment with cyclo(87–99)[Arg^91^, Ala^96^]MBP_87–99_ provides lasting protection against MBP_72–85_-induced EAE.

## 3. Discussion

Cyclic analogues of altered myelin peptide auto-antigens are known to inhibit the development of EAE induced by a different peptide auto-antigen, when co-administered with the immunizing antigen [24,26]. However, this experimental design confounds interpretation of the mechanism of disease inhibition. It is unclear whether cyclic APLs inhibit EAE passively by competing with the disease-inducing antigen for MHC class II binding, or actively through the differentiation of independent, disease-modulating T-cell population(s). The three cyclic mutant analogues used here, cyclo(91–99)[Ala^96^]MBP_87–99_, cyclo(87–99)[Ala^91,96^]MBP_87–99_ and cyclo(87–99)[Arg^91^, Ala^96^]MBP_87–99_, all efficiently bind human HLA-DR4, although with lower affinity than linear MBP_87–99_ [25,26]. Peptide-binding studies with rodent MHC class II molecules have not been performed for the cyclic analogues, and the possibility that MBP_87–99_-based analogues compete with MBP_72–85_ antigen for MHC class II binding, when co-administered in Lewis rats, cannot be excluded. Cyclic peptide analogues might also regulate EAE by inducing Th1/Th2 immune deviation or stimulating inducible regulatory T cells (iTreg). Previous studies by our group showed that immunization of SJL mice with cyclo(87–99)[Ala^91,96^]MBP_87–99_ induces T-cell responses characterized by reduced IFN-γ and increased IL-4 production compared to wild-type MBP_87–99_, suggesting that they promote Th2 cell-type responses [27]. However, T-cell differentiation is dominantly directed by the cytokine milieu and peptide-MHC levels at the time of T-cell activation [31], and EAE immunization protocols purposely induce high level production of T-cell polarization cytokines IL-12 and IL-23 by local antigen-presenting cells, which in turn promote the development of pathogenic Th1 and Th17 cells, respectively [32,33,34]. It is therefore unclear whether co-administration of cyclic MBP_87–99_ analogues and MBP_72–85_ agonist under EAE-inducing conditions [24,26], would permit the differentiation of Th2 cells or iTreg sufficiently to ameliorate disease.

To further investigate the mechanism of disease modulation by three cyclic mutant analogues, cyclo(91–99)[Ala^96^]MBP_87–99_, cyclo(87–99)[Ala^91,96^]MBP_87–99_ and cyclo(87–99)[Arg^91^, Ala^96^]MBP_87–99_, in the current study we administered them in the MBP_72–85_-EAE model in Lewis rats using prophylactic (vaccination) and early therapeutic protocols. In this way, we separated the administration of cyclic analogues from that of the disease-inducing agonist in a temporal manner, so that effects due to possible MHC class II binding competition and immune deviation were minimized. We show that all three cyclic analogues ameliorate EAE when administered prophylactically or therapeutically, and that protection is associated with strong T-cell responses to the cyclic analogues coupled with reduced responses to the EAE-inducing antigen MBP_72–85_. Importantly, longitudinal studies in rats receiving prophylactic cyclo(87–99)[Arg^91^, Ala^96^]MBP_87–99_, showed that treatment provided lasting and robust protection against EAE, up to the last time point studied with statistically significant differences (day 35 post-administration). These studies provide the first indication that cyclic peptide analogues based on the MS candidate auto-antigen MBP_87–99_, provide therapeutic and lasting prophylactic protection against EAE through an active mechanism, involving the activation of peptide analogue-specific T cells and memory T-cell formation. It remains to be determined whether the T cells induced by cyclic peptide analogues show characteristics of peripherally-induced regulatory T cells, which are known to actively suppress T-cell responses through mechanisms such as the secretion of immunosuppressive soluble factors like TGF-beta and IL-10, and competition for the T-cell growth factor IL-2 [35].

The development of cyclic peptide analogues modeled on the MS candidate auto-antigen MBP_87–99,_ is a promising alternative approach for APL immunotherapy in MS. Such analogues demonstrate properties that might be relevant for MS immunotherapy and provide an alternative approach to linear APLs. Previously, our group showed that they suppress the proliferation of a CD4^+^ T-cell line derived from a MS patient, stimulate T-cell responses in peripheral blood monocytes from MS patients inducing Th2 responses when used to immunize mice, show good binding to human HLA-DR4, and show improved stability to proteolysis over linear analogues [24,25,26,27]. Furthermore, we show herein that these analogues have the ability to stimulate protective T-cell responses when administered prophylactically or therapeutically in EAE, and induce lasting protection against EAE through an active mechanism. It remains to be determined whether cyclic MBP_87–99_ analogues protect against CNS autoimmunity in the EAE model through the differentiation of iTreg or an alternative immune-regulatory mechanism.

## 4. Materials and Methods

### 4.1. Peptide Synthesis, Cyclization and Analysis

Peptides (Table 2) were synthesized by Fmoc/tBu methodology using the acid-labile 2-chlorotrityl chloride resin (CLTR-Cl) and *N^α^*-Fmoc (9-fluorenylmethyloxycarboxyl)-protected amino acids as previously described [36,37,38]. The cyclization procedure was performed as described previously [24,26]. The final linear and cyclic peptides were purified and identified using semi-preparative, reverse phase high performance liquid chromatography (RP-HPLC) and electron spray ionization mass spectrometry (ESI-MS; Supplementary Materials), respectively. The purity of linear and cyclic peptides was more than 95% as determined by analytical RP-HPLC.

### 4.2. EAE Induction Evaluation and Tissue Sampling

Experiments were performed in female Lewis rats of 12 weeks of age. EAE was induced by s.c. tail base injection of 30 μg of MBP_72–85_ dissolved in 100 μL saline and emulsified in 100 μL complete Freund’s adjuvant (CFA) (Sigma-Aldrich, St. Louis, MO, USA) supplemented with 400 μg of H37Ra *Mycobacterium tuberculosis* (Difco, Detroit, MI, USA). Rats were weighed and assessed daily for clinical signs according to the following criteria: 0, normal; 0.5, weight loss; 1, limp tail; 2, hind limb weakness; 3, hind limb paralysis; 4, forelimb weakness; 5, moribund or dead. Moribund animals were sacrificed. Rats were allowed free access to food and water throughout the experiment. Rats were maintained under conventional conditions in the experimental animal unit of the Hellenic Pasteur Institute. All experimental protocols were evaluated and approved by the Institutional Protocol Evaluation Committee and the national Hellenic authorities (National Health Authority (PKA) licenses EL25BIO011, EL25BIO012 and EL25BIO013) and conformed to EU guidelines.

### 4.3. In Vivo Treatment with Cyclic Peptide Analogues

The cyclic MBP_87–99_ analogues, cyclo(91–99)[Ala^96^]MBP_87–99_, cyclo(87–99)[Ala^91,96^]MBP_87–99_, and cyclo(87–99)[Arg^91^, Ala^96^]MBP_87–99_ (500 μg) were each dissolved in saline and emulsified in 100 μL incomplete Freund’s adjuvant (IFA) (Sigma-Aldrich, St. Louis, MO, USA) and administered to rats by s.c. tail base injection, at a different site from the immunization site, 2 or 21 days before or 2 days after immunization with MBP_72–85_ for the induction of EAE.

### 4.4. T Cell Proliferation Assay

Draining lymph node (DLN) cells and splenocytes were isolated from EAE control and analogue-treated rats 10 days after immunization with MBP_72–85_ and isolated cells were cultured for 72 h in 96-well plates in RPMI 1640 containing 10% heat-inactivated FCS, 50 μM 2-mercaptoethanol (all Invitrogen Life Technologies, Gaithersburg, MD, USA) and increasing concentrations of MBP_72–85_ agonist or cyclo(87–99)[Arg^91^, Ala^96^]MBP_87–99_ analogue. Cells were pulsed with 1 μCi/5 × 10^5^ cells [3H]thymidine (PerkinElmer, Waltham, MA, USA) for the last 16 h of culture, and [3H]thymidine incorporation was measured by liquid scintillation counting (Wallac, Turku, Finland). Results are expressed as stimulation index (ratio between radioactivity counts of cells cultured in the presence of Ag and of cells cultured with medium alone).

### 4.5. Histopathology

Rats were transcardially perfused with ice-cold 4% paraformaldehyde in PBS under deep anaesthesia. CNS tissues were post-fixed in the same fixative overnight at 4 °C and processed for standard histopathological analyses using paraffin sections (3–5 μΜ). Inflammation was visualized by staining with haematoxylin-eosin according to standard procedures [39].

### 4.6. Statistical Analyses

All statistical analyses were performed with SigmaStat 3.5 (Systat Software Inc., San Jose, CA, USA) for Windows. All data are given as mean ± SEM. To determine significant differences between disease in control and treated rats at different time points after immunization (Figure 1 and Figure 4), the Mann–Whitney rank sum test was performed. Student’s *t*-test was used to compare the inflammatory infiltrates (Figure 2) and T cell proliferation responses to MBP_72–85_ and cyclo(87–99)[Arg^91^, Ala^96^]MBP_87–99_ peptide (Figure 3). Values of *p* < 0.05 were considered statistically significant.

## Figures and Tables

**Table 1:** Incidence, mean day of onset and mean maximal score of myelin basic protein 72–85 epitope (MBP_72–85_)-induced experimental autoimmune encephalomyelitis (EAE) in female Lewis rats, nonvaccinated (control) or vaccinated two days before (prophylactic) and two days after (early therapeutic) induction of EAE, with cyclo(87–99)[Arg^91^, Ala^96^]MBP_87–99_, as shown in three independent experiments (experiments 1–3). Data are also shown (experiment 4) from MBP_72–85_-EAE in groups of rats that were treated prophylactically 23 days before immunization. Mean day of onset and mean maximal score are presented as mean ± SEM.

Rats	Incidence	Mean Day of Onset	Mean Maximal Score
*Expt1*			
Control	3/3 (100%)	11.00 ± 1.08	2.60 ± 0.27
Prophylactic vac d − 2	1/3 (33.3%)	15.00	1.00 ± 0.27
Therapeutic vac d + 2	1/3 (33.3%)	12.00	1.00 ± 0.27
*Expt2*			
Control	6/6 (100%)	8.16 ± 0.15	3.00 ± 0.35
Prophylactic vac d − 2	4/6 (66.6%)	9.25 ± 0.45	0.41 ± 0.14
Therapeutic vac d + 2	5/6 (83.4%)	10.40 ± 1.25	1.25 ± 0.32
*Expt3*			
Control	6/6 (100%)	11.30 ± 0.45	2.60 ± 0.34
Prophylactic vac d − 2	5/6 (83.4%)	12.00 ± 0.32	1.58 ± 0.41
*Expt4*			
Control	4/4 (100%)	9.30 ± 0.27	3.33 ± 0.13
Prophylactic vac d − 23	4/4 (100%)	12.75 ± 0.41	1.87 ± 0.44

**Table 2:** Sequence of studied linear and cyclic myelin basic protein (MBP) peptide analogues.

a/a	Peptide Sequence	Short Name
1	L^72^PQKSQRSQDENPV^85^ (guinea pig)	MBP_72–85_
2	V^87^HFFKNIVTPRTP^99^	MBP_87–99_
3	V^87^HFFA^91^NIVTA^96^RTP^99^	cyclo(87–99)[Ala^91,96^]MBP_87–99_
4	V^87^HFFKNIVTA^96^RTP^99^	cyclo(91–99)[Ala^96^]MBP_87–99_
5	V^87^HFFR^91^NIVTA^96^RTP^99^	cyclo(87–99)[Arg^91^, Ala^96^]MBP_87–99_

## Supplementary Materials

The supplementary materials are available online.
