# Supplementary material for: A Cyclic Altered Peptide Analogue Based on Myelin Basic Protein 87–99 Provides Lasting Prophylactic and Therapeutic Protection Against Acute Experimental Autoimmune Encephalomyelitis
Source: Molecules. 2018 Jan 31;23(2):304. doi: 10.3390/molecules23020304 (PMC6017753; doi:10.3390/molecules23020304)
Supplement: Supplementary file 1 [file molecules-23-00304-s001.pdf]

Supplementary Material

A cyclic altered peptide analogue based on myelin basic protein 87-99 provides lasting prophylactic and therapeutic protection against acute experimental autoimmune encephalomyelitis

Mary Emmanouil[1], Vivian Tseveleki[2], Iro Triantafyllakou[3], Agathi Nteli[4], Theodore Tselios[5,*] and Lesley Probert[6,*]

[1]Laboratory of Molecular Genetics, Hellenic Pasteur Institute, 11521 Athens, Greece; emmanouilm@pasteur.gr

[2]Laboratory of Molecular Genetics, Hellenic Pasteur Institute, 11521 Athens, Greece;tseveleki@gmail.com

[3]Department of Chemistry, University of Patras, 26504 Patras, Greece; irotriant@gmail.com

[4]Department of Chemistry, University of Patras, 26504 Patras, Greece; anteli@upatras.gr

[5]Department of Chemistry, University of Patras, 26504 Patras, Greece; ttselios@upatras.gr

[6]Laboratory of Molecular Genetics, Hellenic Pasteur Institute, 11521 Athens, Greece; lesley.probert@gmail.com

*Correspondence: ttselios@upatras.gr; Tel.: +30-2610997905 and lesley.probert@gmail.com; Tel: +30-2106478866

Table of contents

ESI MS data **S1-S2**

cyclo(91-99)[Ala^{96}]MBP_{87-99} S1

cyclo(87-99)[$Ala^{91,96}$]MBP_{87-99} S2

cyclo(91-99)[Ala^{96}]MBP$_{87\text{-}99}$

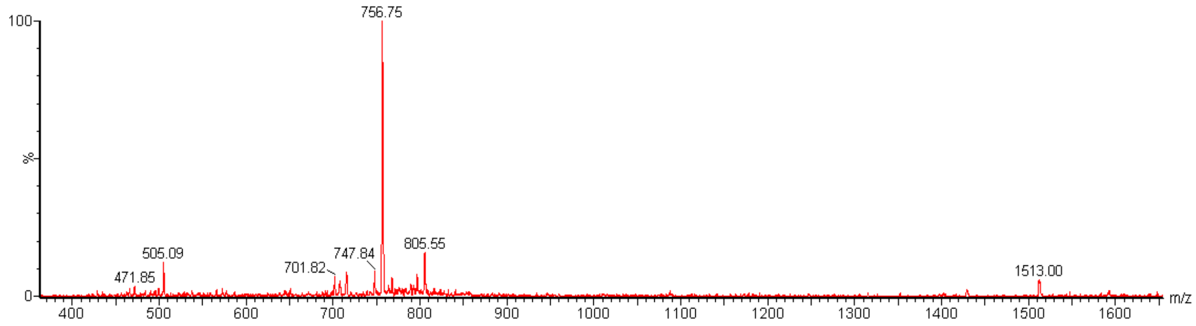

Figure S1: Electrospray ionization mass spectrometry (ESI-MS) spectrum of synthesized analogue cyclo(91-99)[Ala^{96}]MBP$_{87\text{-}99}$. $M_{theoretical}$: 1511.77; $(M+2H^+)/2_{theoretical}$: 756.89. The ESI-MS experiment was performed on a Micromass ZQ Electrospray Platform coupled with a MassLynx 4.1 data system.

cyclo(87-99)[Ala^{91,96}]MBP$_{87\text{-}99}$

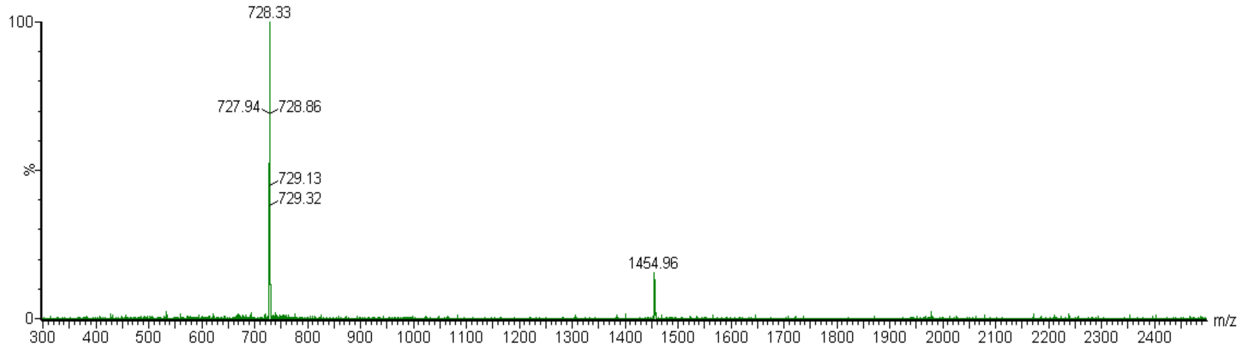

Figure S2: Electrospray ionization mass spectrometry (ESI-MS) spectrum of synthesized analogue cyclo(87-99)[Ala^{91,96}]MBP$_{87\text{-}99}$. $M_{theoretical}$: 1454.67; $(M+2H^+)/2_{theoretical}$: 728.36. The ESI-MS experiment was performed on a Micromass ZQ Electrospray Platform coupled with a MassLynx 4.1 data system.
